# HTLV-1 and HIV-2 Infection Are Associated with Increased Mortality in a Rural West African Community

**DOI:** 10.1371/journal.pone.0029026

**Published:** 2011-12-14

**Authors:** Carla van Tienen, Maarten Schim van der Loeff, Ingrid Peterson, Matthew Cotten, Sören Andersson, Birgitta Holmgren, Tim Vincent, Thushan de Silva, Sarah Rowland-Jones, Peter Aaby, Hilton Whittle

**Affiliations:** 1 Medical Research Council, Fajara, The Gambia; 2 Municipal Health Service and Academic Medical Centre, Amsterdam, The Netherlands; 3 Swedish Institute of Infectious Disease Control, Stockholm, Sweden; 4 Department of Laboratory Medicine, Malmö, Lund University, Lund, Sweden; 5 University College London Centre for Medical Molecular Virology, Division of Infection and Immunity, University College London, London, United Kingdom; 6 Weatherall Institute of Molecular Medicine, Human Immunology Unit, John Radcliffe Hospital, Oxford, United Kingdom; 7 Projecto de Saúde de Bandim, Indepth Network, Bissau, Guinea-Bissau; Emory University School of Medicine, United States of America

## Abstract

**Background:**

Survival of people with HIV-2 and HTLV-1 infection is better than that of HIV-1 infected people, but long-term follow-up data are rare. We compared mortality rates of HIV-1, HIV-2, and HTLV-1 infected subjects with those of retrovirus-uninfected people in a rural community in Guinea-Bissau.

**Methods:**

In 1990, 1997 and 2007, adult residents (aged ≥15 years) were interviewed, a blood sample was drawn and retroviral status was determined. An annual census was used to ascertain the vital status of all subjects. Cox regression analysis was used to estimate mortality hazard ratios (HR), comparing retrovirus-infected versus uninfected people.

**Results:**

A total of 5376 subjects were included; 197 with HIV-1, 424 with HIV-2 and 325 with HTLV-1 infection. The median follow-up time was 10.9 years (range 0.0–20.3). The crude mortality rates were 9.6 per 100 person-years of observation (95% confidence interval 7.1-12.9) for HIV-1, 4.1 (3.4–5.0) for HIV-2, 3.6 (2.9–4.6) for HTLV-1, and 1.6 (1.5–1.8) for retrovirus-negative subjects. The HR comparing the mortality rate of infected to that of uninfected subjects varied significantly with age. The adjusted HR for HIV-1 infection varied from 4.0 in the oldest age group (≥60 years) to 12.7 in the youngest (15–29 years). The HR for HIV-2 infection varied from 1.2 (oldest) to 9.1 (youngest), and for HTLV-1 infection from 1.2 (oldest) to 3.8 (youngest).

**Conclusions:**

HTLV-1 infection is associated with significantly increased mortality. The mortality rate of HIV-2 infection, although lower than that of HIV-1 infection, is also increased, especially among young people.

## Introduction

HIV-1, HIV-2 and Human T-cell Lymphotropic Virus type 1 (HTLV-1) are all endemic in Guinea-Bissau. While HIV-1 in the absence of treatment leads to AIDS and death in the vast majority of infected individuals, only 50% of people with HIV-2 infection are estimated to progress to AIDS and death [1]. HTLV-1 causes a lethal form of leukaemia, Adult T-cell Leukemia, and a debilitating neurological syndrome, Tropical Spastic Paresis, in up to 5% of infected individuals and is associated with a number of infectious and inflammatory conditions (reviewed in [2]). Reports on mortality associated with HTLV-1 infection vary and do not always find significantly increased mortality rates compared to uninfected people (mortality rate ratios ranging from 1.1 to 1.9) [3], [4], [5], [6].

Dual infection with HIV-1 and HIV-2 is relatively common in West Africa (reviewed in [7]) and most studies have found a similar mortality in HIV-1 single infections and HIV-1/HIv-2 dual infections, suggesting HIV-1 is the driving force in disease progression. This was based on studies in clinical settings [8], [9], [10] and only one community based study among older individuals [5].

Dual infection with HIV-1 and HTLV-1 has been described mainly in African and South-American countries and high-risk patient groups (commercial sex workers and iv drug users) in Europe [11], [12], [13]. Whether HTLV-1 co-infection leads to a faster disease progression in HIV infection and what its effect is on mortality remains unclear, because most studies were cross-sectional and lacked control groups of singly HTLV-1 infected people (reviewed in [14]). In Guinea-Bissau, HTLV-1/HIV-2 dual infection is relatively common (12–20% of HIV-2 infected people) [15], [16], [17], [18]. Two studies examined the effect of co-infection and did not observe an increased mortality in HTLV-1/HIV-2 co-infected individuals compared to HIV-2 singly infected people [5], [19], although one study did find a higher mortality in HTLV-1/HIV-2 co-infected individuals with pulmonary tuberculosis as compared to HIV-2 singly infected individuals [20]. A report from the population in which the current study was performed found a higher CD4% and a lower HIV-2 viral load in HTLV-1 infected compared to HTLV-1 uninfected people, suggesting a potential beneficial effect [19].

Caió, the community described in this study, has had the highest prevalence of HIV-2 (8%) in adults world-wide, has an increasing HIV-1 prevalence (currently 4%) and the highest HTLV-1 prevalence (5%) in adults in Guinea-Bissau [15], [21]. We examined the effects of retroviral single and dual infections on mortality in a community based cohort in rural Guinea-Bissau over a 20-year period.

## Methods

### Ethics Statement

All studies were approved by the Gambia Government/MRC Laboratories Joint Ethics Committee and by the Ministry of Health of Guinea-Bissau. Informed consent was obtained from all the study participants. Local community spokesmen, representatives of the Guinea-Bissau government and the local nurses and physicians were always first informed about the planned studies, after which village meetings were held to inform all Caio residents. Prior to 2003 verbal informed consent was obtained from all study participants. From 2003 onwards, written informed consent from all study participants was obtained as required by the Gambian Ethics Committee.

### Study area and subjects

The study was conducted in a rural area in North-western Guinea-Bissau, consisting of ten small settlements in a forest. The population consists mostly of people from the Manjako ethnic group. The main agricultural products are cashew nuts, cashew wine, palm wine, palm oil, and rice. Since 1989 a demographic database of the population has been maintained. Field workers visit all compounds at least once a year to record changes (deaths, births, moves, emigration, and immigration). When a person has died in the compound, a basic questionnaire concerning the conditions of the person prior to his/her death, is administered to the closest relative by the fieldworker. Every village has a village reporter who reports on vital events. Many men work and live in large towns in the sub-region and in Europe, mainly in Portugal and France. Because family links are very strong, news about deaths abroad of people originating from the study area reliably reaches the community. Also, a special ceremony is held after a death of an adult which lasts 1–2 days and therefore we assume the majority of deaths in the community are recorded accurately in the census. More adult men than women spend time away, creating a low men-women ratio. People who keep a room in their house or compound, and who are expected to return, are considered residents.

The current analysis comprises data of three population surveys among adults carried out in 1989–1991, 1996–1998 and 2006–2007, in which approximately 75% of all the residents were included [15], [21]. These study rounds will be referred to as 1990, 1997 and 2007 hereafter. HIV-positive and -negative individuals identified in these surveys were then invited to join a cohort (matched by sex, age and area of living) which was initiated in 1991; members of this cohort were re-examined in 1996, 2003 and 2006.

Cohort members have free access to medication and medical care provided by the physician who is based permanently at the research project run by the Medical Research Council and the Bandim Health Project in Caio. The project building houses a laboratory, small pharmacy, consultation room and administrative offices. Anti-retroviral treatment for HIV became available in 2007 as part of the national AIDS program, after completion of the 2007 survey.

### Laboratory methods: HIV testing

HIV testing methods have been described [21], [22]. In brief, in the 1990 survey the HIV diagnoses were determined by serology (Murex Diagnostics, Dartford, UK) and most of these samples were confirmed by PCR in a follow-up study in 1991 [22]. In the 1997 survey the following algorithm was used: plasma samples were first screened by ELISA (Murex Diagnostics, Dartford, UK), reactive samples were then tested by ELISAs that are mono-specific for HIV-1 and HIV-2 (Wellcozyme 1, Wellcozyme II and ICEHIV-2, Murex Diagnostics, Dartford, UK) and by a synthetic peptide-based assay (Pepti-Lav, Pasteur, France). Dually reactive and indeterminate samples underwent PCR for confirmation [23]. In the 2007 survey, plasma samples were first screened by ELISA (Murex Diagnostics, Dartford, UK). Subsequently, HIV-1 or HIV-2 confirmation was obtained using a synthetic peptide-based assay (Hexagon, Human, Germany). Dually reactive and indeterminate samples were subjected to a different synthetic peptide-based assay (Pepti-Lav 1-2, Sanofi Diagnostics, France). Indeterminate results were resolved using HIV-1 and HIV-2-specific PCR [21].

### Laboratory methods: HTLV testing

For the 1990 survey, two different screening ELISAs (Organon Teknika, Boxtel, The Netherlands and Murex HTLV-1/2, Abbott/Murex Diagnostics, Dartford, UK) were used and positive samples were confirmed by PCR and/or Western Blot as described [18], [19]. For the 1997 and 2007 survey, an ELISA (Murex I/II, Abbott Murex Diagnostics, Dartford, UK) was used to screen all samples. Reactive samples were retested with the same ELISA (Murex I/II, Abbott Murex Diagnostics, Dartford, UK) and were confirmed by PCR using primer pairs derived from the *tax/rex* gene for the 1997 samples [24] and by nested PCR using primers targeted to either the *gag* p24 open reading frame or to the *tax* gene for the 2007 samples [15]. These primers were specific for HTLV-1. As a control for DNA quality, all samples testing negative for both p24 and *tax* PCR were tested for presence of human DNA, using primers against the human beta-2-microglobulin gene and all were positive. Subjects were considered HTLV-1 infected if at least one of the 2 ELISAs was reactive and either PCR or Western Blot was positive.

### Statistical Methods

Analysis was done using Stata 11 (Stata Corporation, College station, TX, USA). Differences in categorical variables were examined by chi-square test or Fisher's exact test. When age was treated as a continuous variable, differences between groups were examined by Wilcoxon's ranksum test.

The information collected during the first survey at which a person participated in the study (this could be either in 1990, 1997 or 2007), was used as baseline information.

Survival probability was examined by Kaplan-Meier (KM) graphs. The follow-up time started on the date when subjects were first enrolled with interview and blood sample (i.e. in the survey in 1990, 1997 or 2007) and ended on the date of death, or on the date of moving out of the area with no more information being available for the subject ( =  loss to follow-up) or on the date they were last known to be alive during the last census (2009), or on the date they were tested positive for a different retroviral infection, or on the date subjects started anti-retroviral treatment (ART), whichever came first. After seroconversion, the observation time would start again from time 0. This means that subjects who seroconverted contributed to more than one retroviral group.

Cox regression was used to calculate mortality Hazard Ratios (HR). The proportional hazard's assumption was assessed graphically and by statistical testing (Schoenfeld residuals). Age was used as a categorical variable with 4 age groups. Age and retroviral status were treated as time-varying variables. In the multivariable analysis, all baseline variables from the univariate analysis were entered into an initial model. Variables were then dropped one by one if they were not significant predictors of mortality and if their omission did not change the HR of the HIV and HTLV-1 status variables in a substantial way (≥10%). Models were compared with likelihood ratio tests (LRT) and the Wald test was used to compare model parameters (Hazard Ratios).

The STROBE guidelines were used to report the findings of this study in this paper [25].

## Results

### Subjects

In total, 5,822 subjects provided a blood sample in one or more of the three study rounds (in 1990, 1997 and 2007). Of these, 446 (8%) subjects were excluded from the analysis due to: age <15 years (n = 30, 7%), no re-identification of subject possible (n = 66, 15%), no census follow-up after a subject had participated for the first time in the 2007 study round (n = 105, 24%), and indeterminate or missing test results due to insufficient blood volume for testing of all retroviral infections (n = 245, 55%). The total number of subjects that had a final HIV and HTLV-1 result, at one or more study rounds, was 5,376 (Table 1).

**Table 1:** Baseline characteristics of 5376 adults by retroviral status, Caió, 1989-2009.

	HTLV-1 negative				HTLV-1 positive			
	HIV neg^a^	HIV-1 pos	HIV-2 pos	HIV-1/2 pos^b^	HIV neg	HIV-2 pos	HIV-1 or HIV-1/2 pos^c^	Total
**Total number**	4,797	55	235	14	200	61	14	5376
**Number** **female (%)**	2,801 (58)	30 (55)	151 (64)	12 (86)*	131 (66)*	54 (89)*	12 (86)*	3191 (59)
**Median age** (IQR)	25 (20–43)	31 (25–42)	44 (34–58)*	47 (35–60)*	42 (23–64)*	59 (45–67)*	48 (42–54)*	27 (20–47)
**Age group (%)**								
15–29	2,878 (60)	26 (47)*	40 (17)*	1 (7)*	74 (37)*	3 (5)*	–	3,022 (56)
30–44	785 (16)	20 (36)*	80 (34)*	6 (43)*	37 (19)*	12 (20)*	5 (36)*	945 (18)
45–59	502 (10)	5 (9)*	64 (27)*	3 (21)*	29 (15)*	17 (28)*	8 (57)*	628 (12)
60-max	632 (13)	4 (7)*	51 (22)*	4 (29)*	60 (30)*	29 (48)*	1 (7)*	781 (15)
**Ethnic group Manjako (%)**	4,561 (95)	49 (89)	218 (93)	12 (86)	189 (95)	60 (98)	14 (100)	5103 (95)
**Central zone of living (%)**	3,156 (66)	43 (78)*	184 (79)*	10 (71)	144 (73)	50 (82)*	10 (71)	3597 (67)
**Marital status (%)**								
Single	2,223 (46)	18 (33)*	36 (15)*	1 (7)*	62 (31)*	4 (7)*	2 (14)*	2346 (44)
Married	2,314 (48)	29 (53)*	186 (71)*	5 (36)*	102 (51)*	30 (49)*	9 (64)*	2657 (49)
Widowed	201 (4)	6 (11)*	21 (9)*	6 (43)*	28 (14)*	21 (34)*	2 (14)*	285 (5)
Divorced	54 (1)	2 (4)*	10 (4)*	1 (7)*	8 (4)*	6 (10)*	1 (7)*	82 (2)
**Number with only baseline sample (%)** d	2601 (54)	34 (62)	87 (37)*	9 (64)*	111 (56)	26 (43)	9 (64)	2877 (54)

Neg, negative; pos, positive; IQR, interquartile range; ranksum test for age, chi square for proportions of infected versus HIV and HTLV-1 uninfected individuals (

* = significant at 5% level).

a 5 subjects had missing data for marital status.

b 1 subject had missing data for marital status.

c 5 subjects were HTLV-1/HIV-1 infected and 9 were HTLV-1/HIV-1/2 infected.

d please see Van Tienen et al. JAIDS 2010 for the differences between subjects with and without a follow-up sample.

The baseline characteristics are listed in Table 1. Among the HTLV-1 uninfected, at baseline there were 55 (1%) HIV-1, 235 (4%) HIV-2 and 14 (0.3%) HIV-1/2 dually infected subjects. Among the HTLV-1 infected, 200 (4%) subjects were singly HTLV-1 infected, 61 (1%) subjects were HTLV-1/HIV-2 dually infected and 14 (0.3%) were HTLV-1/HIV-1 dually or HTLV-1/HIV-1/2 triply infected. The median age at enrolment was 27 years (interquartile range, 20-47). Most subjects were Manjako (n = 5,103; 95%), which was similar for the various retroviral infected groups. Individuals infected with HIV-2 and/or HTLV-1 or HIV-1 and HIV-2, were older and a higher percentage was female compared to the uninfected group. Retroviral infected subjects were less often single and more often divorced or widowed compared to the uninfected subjects.

### Follow-up

The follow-up details by retroviral status are listed in Table 2. During follow-up, 40 subjects acquired HTLV-1 and 169 subjects acquired HIV-1 and/or HIV-2. These seroconverters contribute to more than one column in Table 2. The median observation time was 10.9 years (range 4 days – 20.3 years). A total of 177 (3%) people were lost to follow-up, 24 (0.4%) people started anti-retroviral treatment (ART) and a total of 1089 (20%) persons died. The overall mortality rate was 2.0 (95% CI 1.9–2.1) per 100 person years of observation.

**Table 2:** Follow-up of subjects included in the survival analysis by retroviral status, Caió, 1989-2009.

	HTLV-1 negative				HTLV-1 positive			
	HIV neg	HIV-1 pos	HIV-2 pos	HIV-1/2 pos	HIV neg	HIV-2 pos	HIV-1 or HIV-1/2 pos^a^	Total
**Number of subjects** b	4,797	117	285	53	229	69	27	5376
**Lost to follow-up (%)**	161 (3)	-	8 (3)	-	6 (3)	2 (3)	-	177 (3)
**Started ART (%)**	-	8 (7)	12 (4)	4 (8)	-	-	-	24 (0.4)
**Number of deaths (%)**	786 (16)	43 (40)	111 (39)	24 (45)	73 (32)	33 (48)	19 (70)	1,089 (20)
**Pyo**	47,986	450	2,680	272	2,003	568	113	54,071
**MR per 100 pyo** **(95% CI)**	1.6 (1.5–1.8)	9.6 (7.1–12.9)	4.1 (3.4–5.0)	8.8 (5.9–13.1)	3.6 (2.9–4.6)	5.8 (4.1–8.2)	16.9 (10.8–26.4)	2.0 (1.9–2.1)
**Crude MRR (95% CI)**	1	5.8 (4.3–7.9)	2.5 (2.1–3.1)	5.4 (3.6–8.1)	2.2 (1.8–2.8)	3.5 (2.5–5.0)	9.8 (6.2–15.5)	-

Neg, negative; pos, positive; ART, Anti-retroviral treatment; Pyo, person-years of observation; MR, mortality rate; MRR, mortality rate ratio; CI, confidence interval.

a 10 subjects were HTLV-1/HIV-1 infected (of which 5 died) and 17 were HTLV-1/HIV-1/2 infected (of which 14 died).

b subjects that seroconverted contribute to different columns.

The lay reports of symptoms and conditions before death are given in Table 3. Symptoms that may be associated with immunosuppression, such as diarrhea, cough or respiratory problems, high fever and vomiting were more common in HIV infected than HIV uninfected individuals that died, especially among HIV-1 and HIV-1/2 infected people.

**Table 3:** Signs and symptoms related to 1089 deaths among adults by retroviral status, Caió, 1989–2009.

	HTLV-1 negative				HTLV-1 positive		
Symptoms/signs^a^	HIV neg	HIV-1 pos	HIV-2 pos	HIV-1/2 pos	HIV neg	HIV-2 pos	HIV-1 or HIV1/2 pos
**Total number of deaths**	786	43	111	24	73	33	19
**Number of women**	415 (52.8)	21 (48.8)	57 (51.4)	21 (87.5)*	45 (61.6)	30 (90.9)*	17 (89.5)*
**Diarrhea**	23 (3.0)	8 (18.6)*	6 (5.5)	7 (29.2)*	4 (5.5)	2 (6.1)	1 (5.3)
**Cough/respiratory problems**	29 (3.7)	9 (20.9)*	10 (9.0)*	5 (20.8)*	3 (4.1)	5 (15.2)*	1 (5.3)
**High fever**	84 (10.8)	14 (32.6)*	17 (15.5)	8 (33.3)*	13 (17.8)	4 (12.1)	-
**Vomiting**	17 (2.2)	5 (11.6)*	6 (5.5)*	3 (12.5)*	4 (5.5)	3 (9.1)*	-
**Paralysis**	20 (2.6)	1 (2.3)	3 (2.7)	-	1 (1.4)	1 (3.0)	-
**Hospitalized**	179 (23.0)	25 (58.1)*	38 (34.6)*	9 (37.5)	16 (21.9)	8 (24.2)	8 (42.1)*
**Accident** b	34 (4.3)	-	3 (2.7)	-	3 (4.1)	1 (3.0)	-

Neg, negative; pos, positive; chi-square for proportions of infected versus uninfected individuals (*  =  significant at 5% level).

a please note that individuals could have more than one sign or symptom (so numbers of deaths do not match the total numbers of symptoms and signs).

b drowning, snake bite, fall from/hit by tree, car accident, suicide.

### Mortality proportional hazard analysis

The univariate HR for mortality was significantly increased for all retroviral infection groups (p<0.001), compared to the uninfected group (Table 4). Differences in sex, age group, marital status and calendar period significantly influenced the HR. Ethnicity and zone of living were not associated with increased mortality. When 25 HIV-2 positive people with a history of blood transfusion were excluded, because this may be related to a worse health condition or a higher viral load, this did not change the HR for HIV-2.

**Table 4:** Univariate Mortality Hazard Ratios of adults, Caió, 1989–2009.

	Mortality Hazard Ratio (95% CI)	p-value
**HIV/HTLV-1 status**		
HIV/HTLV-1 neg	1	<0.001
HTLV-1 negative:		
HIV-1 pos	7.3 (5.3–10.0)	
HIV-2 pos	2.6 (2.1–3.2)	
HIV-1/2 dual pos	6.6 (4.4–9.9)	
HTLV-1 positive:		
HIV neg	2.3 (1.8–2.9)	
HIV-2 pos	3.7 (2.6–5.3)	
HIV-1 or HIV-1/2	12.4 (7.8–19.6)	
**Sex**		
Male	1	<0.001
Female	0.7 (0.7–0.8)	
**Age group**		
15–29	1	<0.001
30–44	2.6 (2.0–3.4)	
45–59	4.8 (3.7–6.4)	
60-max	15.2 (11.9–19.5)	
**Marital status**		
Single	1	<0.001
Married	2.0 (1.7–2.4)	
Divorced	5.2 (4.2–6.3)	
Widowed	6.6 (5.2–8.3)	
**Ethnic group**		
Manjako	1	0.1
Other	0.8 (0.5–1.1)	
**Zone of living**		
Central	1	0.4
Periphery	0.9 (0.8–1.1)	
**Time period**		
1989–1993	1	0.05
1994–1998	1.4 (1.1–1.8)	
1999–2003	1.2 (0.9–1.5)	
2004–2009	1.1 (0.8–1.4)	

CI, confidence interval; neg, negative; pos, positive.

**Figure 1:**
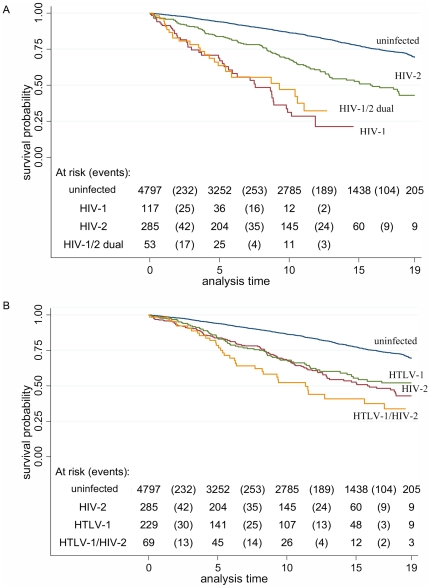
A. Kaplan-Meier graph of survival probability by HIV-status for subjects from Caió, 1989–2009 (HTLV-1 infected subjects excluded). B. Kaplan-Meier graph of survival probability by HTLV-1 and HIV-2 infection status for subjects from Caió, 1989–2009 (HIV-1 and HIV-1/2 infected subjects excluded).

Differences in survival between retroviral infection groups can be observed in Figures 1a and 1b. The survival probability differed significantly between each retroviral infection group and the uninfected group (logrank test, p<0.001), between HIV-1 and HIV-2 (p<0.001), between HIV-1/2 dually and HIV-2 (p<0.001) and between HTLV-1/HIV-2 dually and HTLV-1 infected subjects (p = 0.03).

In the multivariable model, HIV status, HTLV-1 status, sex, age and marital status remained significantly associated with mortality and were included. All possible interactions between the two main exposures, HIV and HTLV-1 status, and the other variables were tested. Strong and significant interactions were found between HIV and age (LRT, p<0.001) and between HTLV-1 and age (p = 0.01). HRs are reported stratified by age (Table 5). Strikingly, in HIV-2 and HTLV-1 infection, the HRs were highest (and highly significant) in the youngest age category (15-29 years), 9.1 and 3.8 respectively, and decreased with age, to 1.2 and 1.2 in the 60+ age group (both non-significant). Also in HIV-1 and HIV-1/2 dually infected individuals, the lowest HRs were observed in the oldest age group, being 4.0 and 3.3, respectively (p = 0.002 and p<0.001, respectively). The age-stratified HRs of HIV-1 and HIV-1/HIV-2 dual infection were not significantly different (Wald test, p>0.4 for all age groups).

**Table 5:** Adjusted Mortality Hazard Ratios of adults by retroviral infection, stratified by age and sex, Caió, 1989-2009.

		Adjusted Mortality Hazard Ratio (95% CI)		
Age group	N deaths/N subjects^a^	Men and women^b^	Men^c^	Women^c^
**HIV-1**				
15–29	4/38	12.7 (4.5–35.6)	-	21.4 (7.1–64.0)
30–44	22/72	13.9 (8.5–22.7)	11.7 (6.1–22.3)	15.4 (7.2–33.2)
45–59	17/37	15.2 (8.6–26.7)	10.9 (4.5–26.1)	19.2 (9.2–40.2)
60-max	5/12	4.0 (1.6–9.7)	3.2 (1.0–10.1)	6.1 (1.5–25.1)
**HIV-2**				
15–29	6/54	9.1 (3.8–21.7)	-	16.6 (6.4–43.3)
30–44	24/145	3.6 (2.3–5.6)	2.6 (1.3–5.2)	4.4 (2.4–8.2)
45–59	38/168	2.5 (1.7–3.6)	2.1 (1.2–3.7)	3.0 (1.8–5.1)
60-max	76/157	1.2 (0.9–1.5)	1.4 (0.9–2.0)	1.1 (0.8–1.5)
**HIV-1/2 dual**				
15–29	1/6	29.2 (3.9–217.0)	-	88.8 (10.7–738.0)
30–44	9/27	10.2 (5.0–20.6)	6.7 (2.1–21.7)	13.7 (5.5–33.9)
45–59	13/37	11.4 (6.1–21.2)	4.7 (0.6–34.4)	15.2 (7.5–30.6)
60-max	15/25	3.3 (1.9–5.5)	-	3.3 (1.9–5.6)
**HTLV-1**				
15–29	7/86	3.8 (1.7–8.5)	4.5 (1.8–11.7)	2.0 (0.4–9.2)
30–44	14/97	2.3 (1.3–4.1)	2.0 (0.6–6.3)	2.2 (1.1–4.5)
45–59	20/101	1.4 (0.9–2.3)	1.5 (0.5–4.0)	1.4 (0.8–2.5)
60-max	84/150	1.2 (0.9–1.5)	0.9 (0.5–1.4)	1.3 (1.0–1.8)

CI, confidence interval.

a Number of deaths among retroviral positive subjects/Number of retroviral positive subjects – NB subjects can contribute to more than one age group because age was treated as a time-varying variable.

b adjusted for all other retroviral infections, marital status, sex.

c adjusted for all other retroviral infections, marital status.

## Discussion

### Key findings

This cohort study has confirmed an increased risk of death among HIV-1, HIV-2 and HTLV-1 infected individuals compared to uninfected individuals in a rural area of Guinea-Bissau. To our knowledge, this is the largest and longest community based study that has examined the effects of infection with these three retroviruses on mortality. Notably, this population had an excellent follow-up and was ART-naïve during the study and therefore the observed mortality reflects the natural effect of these retroviral infections on mortality. The highest mortality was observed in HIV-1 and HIV-1/2 dually infected individuals and the age-stratified HRs were similar in these infection groups. HIV-2 and HTLV-1 infected subjects had a more moderately increased mortality and the highest HR was observed in the youngest subjects.

### HIV related mortality

The more than 10-fold increase in mortality rate of HIV-1, as observed in all age groups except the oldest, is in line with community based studies from other parts of Africa, where similarly increased mortality rates have been observed [26]. It is higher than the 5-fold increase that was found in a study from the capital Bissau, which may be related to the older age and a higher loss to follow-up in the Bissau cohort [5].

This study confirms the increased mortality rate in HIV-2 infection, observed in previous analyses from this study population [27], [28] and other community based studies [5], [29]. Generally, the HR was lower than the HR (3.5) observed in the same cohort in 1991–1993 [28]. In the Bissau cohort, a higher HR was also observed in the first year of follow-up which then decreased during subsequent observation [29]. This could be related to the fact that those subjects that are unable to control the infection will have died, leaving a majority of long-term survivors among the HIV-2 positive subjects, who have a lower HR. The HR declined with increasing age. This is probably due to two factors: a higher background mortality among older individuals and a survival bias of a group of older individuals that are not progressing to disease. In HIV-2 infection, certain individuals control the virus, maintaining low viral loads for prolonged periods of time, and have a normal lifespan [30], while others progress rapidly and contribute to the observed excess mortality. Understanding the biological mechanisms behind this phenomenon could lead to better understanding of HIV control by the immune system and could contribute to vaccine development for both HIV-1 and HIV-2.

The HRs of HIV-1 and HIV-1/2 dually infected subjects were not significantly different from each other in this study. Several studies have observed that mortality rates among HIV-1 singly and HIV-1/2 dually infected individuals are similar [5], [8], [9], [10], suggesting HIV-1 determines the outcome of the infection on mortality. The lower HRs observed in the oldest individuals is probably related to the higher background mortality in this age group.

### HTLV-1 related mortality

An increased mortality rate with HTLV-1 infection was observed. The effect of this infection on mortality was independent from the mortality effect of HIV-2. Thus the increased mortality rate among co-infected people is the result of the increase in mortality due to HIV-2 infection and due to the increase in mortality due to HTLV-1 infection; there are no indications that co-infection leads to a more rapid disease progression of HIV-2. This is in agreement with a study performed in a subset of this population, which found that there was no enhancing effect of HTLV-1 co-infection on the replication of HIV-2 *in vivo* [19]. HTLV-1 is associated with an increased CD4 count, also in HIV-2 infection, but this does not seem to result in a better outcome of the infection [19].

What causes this increased mortality among usually asymptomatic carriers of HTLV-1? A case-control study performed in Caió showed a prevalence of 7.1% of probable Tropical Spastic Paresis in HTLV-1 infected subjects [31] and the incidence of Adult T-cell Leukemia is not known. A number of infectious and inflammatory conditions have been associated with HTLV-1 infection in the past decades, that may be related to altered immune responses (reviewed in [2]). Cross-sectional and case-control studies have shown an increase of TB in HTLV-1 infection [32]. From the lay reports of symptoms before death in the current study, there was no indication that cough or respiratory symptoms were more frequent among HTLV-1 single infected individuals, but that it was elevated among HTLV-1/HIV-2 dually infected individuals. This would be in line with a study from Bissau where a higher prevalence of HTLV-1 was found among HIV-positive TB patients, but not among HIV-negative TB patients [33], but would not explain the independent increased risk of death among HTLV-1 single infected individuals observed in this study. No formal studies have been conducted in Caió to determine the incidence of TB and the death reports were entirely done by lay field workers and therefore need to be interpreted with caution.

### Gender specific mortality

HIV-2 single and dual infections are more common among women than men (reviewed in [34]) and the prevalence increases with age. One reason for this observed difference may be a gender specific difference in mortality. In the current study we found that in our total population the crude (or unadjusted) mortality was significantly lower among women than men (MRR 0.7, CI 0.7–0.8). This is most likely related to the fact that non-HIV related mortality is usually higher in men than in women [35]. In the multivariable analysis however, there was no modifying effect of sex on mortality, i.e. there was no interaction between sex and mortality. Hence, there is no higher mortality among men that could explain the difference in retroviral prevalence. Increased sexual risk behavior has not been proven to explain this difference either [36]. Other possible explanations are an increased susceptibility of older women due to changes in the vaginal mucosa [34], [37].

### Public health implications

This study was performed in a treatment-naïve population, where HIV and HTLV-1 infection are endemic. Although antiretroviral treatment became available in 2005 in the capital, the treatment in Caió only started in 2007. HIV/HTLV-1 co-infection is associated with increased CD4 counts [14], [19], [20], which complicates the decision when to start treatment. The intermittent antiretroviral drug supply also poses many difficulties in the treatment of HIV patients in Guinea-Bissau [38]. Although national programs are in place for prevention of transmission, this is mainly limited to the capital and is not fully operational in rural areas like Caió. Despite local efforts to educate the Caió community about HIV - e.g. through village meetings, free distribution of condoms, NGO prevention programs - HIV-1 increased during the study period [21]. It is crucial that public health efforts are made to prevent HIV and HTLV-1 transmission and to give appropriate care to infected individuals.

### Limitations

Due to the long intervals between the study rounds, the time of infection of the subjects in this study were unknown. A follow-up sample was available for 46% of the subjects. In this group, seroconversions could be established, but in people without subsequent samples unchanged retroviral status was assumed in the analysis. This will have led to an underestimation of the HRs; for example, some subjects that were HIV-negative at baseline will have seroconverted and died while contributing to the uninfected group. Among the HIV-2 (and possibly HTLV-1) infected individuals the incidence of HIV-1 is higher than among uninfected individuals [23]. Thus it is possible that the mortality among HIV-2 (and HTLV-1) infected persons is overestimated in this study, although the number of persons with a follow-up sample(s) among HIV-2 infected people was higher (63%) than among the other groups (Table 1).

### Conclusion

To our knowledge, this is the largest community cohort study to examine the combined effects of HIV-1, HIV-2 and HTLV-1 on mortality. The occurrence and size of the excess mortality in both HIV infections can be explained by AIDS and is in line with other studies. The excess mortality associated with HTLV-1 reported in other studies is confirmed in this study, but its causes remain to be elucidated.
